# Rhamnolipid Micellization and Adsorption Properties

**DOI:** 10.3390/ijms231911090

**Published:** 2022-09-21

**Authors:** Yi Zhang, Tess L. Placek, Ruksana Jahan, Paschalis Alexandridis, Marina Tsianou

**Affiliations:** Department of Chemical and Biological Engineering, University at Buffalo, The State University of New York (SUNY), Buffalo, NY 14260-4200, USA

**Keywords:** biosurfactant, green surfactant, rhamnolipid, self-assembly, formulation, bioremediation, sustainability

## Abstract

Biosurfactants are naturally occurring amphiphiles that are being actively pursued as alternatives to synthetic surfactants in cleaning, personal care, and cosmetic products. On the basis of their ability to mobilize and disperse hydrocarbons, biosurfactants are also involved in the bioremediation of oil spills. Rhamnolipids are low molecular weight glycolipid biosurfactants that consist of a mono- or di-rhamnose head group and a hydrocarbon fatty acid chain. We examine here the micellization of purified mono-rhamnolipids and di-rhamnolipids in aqueous solutions and their adsorption on model solid surfaces. Rhamnolipid micellization in water is endothermic; the CMC (critical micellization concentration) of di-rhamnolipid is lower than that of mono-rhamnolipid, and both CMCs decrease upon NaCl addition. Rhamnolipid adsorption on gold surface is mostly reversible and the adsorbed layer is rigid. A better understanding of biosurfactant self-assembly and adsorption properties is important for their utilization in consumer products and environmental applications.

## 1. Introduction

Biosurfactants are molecules with surface-active properties produced by microorganisms (bacteria, fungi, yeasts) [1,2]. Biosurfactants usually have hydrophobic groups of proteins and/or peptides or carbon chains, and hydrophilic groups comprising esters, hydroxyl, phosphate, carboxyl, or carbohydrates [3].

Surfactants play an important role in numerous processes and products for rheological modification [4,5], surface modification [6,7], emulsions [8,9], dispersions [10,11], material synthesis [12,13], drug delivery [14], etc. Accordingly, biosurfactants have a wide range of potential applications, including detergents in household and agriculture products, solubilizers in pharmaceuticals, wetting and penetrating agents, emulsifiers, dispersants in paints, wetting and thickening agents [15,16]. The application of biosurfactants in personal care products and cosmetics is trending due to their low toxicity, excellent moisturizing ability, and skin compatibility [17]. In enhanced oil recovery, oil spill cleanup, and bioremediation of hydrophobic pollutants, biosurfactants are promising substitutes to the currently utilized synthetic surfactants [18,19,20]. Biosurfactants can be very selective and effective under many conditions, and only small quantities are required for the oil recovery or cleaning; further, the whole cell broth can be utilized without a complicated purification process [21,22,23,24].

Among the various types of biosurfactants, the most widely studied class are the glycolipids, and rhamnolipid is the most extensively studied glycolipid biosurfactant [25,26]. Rhamnolipids find application in food, health and beauty products, pharmaceutical and therapeutics, detergents and cleaners, enhanced oil recovery, bioremediation, etc. [26,27,28,29]. Compared with other biosurfactants, the number of patents on rhamnolipids is the highest [27]. Rhamnolipids are found in commercial products such as hand soaps (MayLu) [30] and biofungicide Zonix^TM^ [31].

Rhamnolipids are produced by several types of microorganisms: the main producing species is the bacterium *Pseudomonas aeruginosa*. The bacterial strains *Nocardiopsis* spp.*, Acinetobacter calcoaceticus, Serratia rubidaea, Burkholderia* spp., and *Enterobacter* spp. Are also able to produce rhamnolipids under suitable conditions [26]. Several industrial waste products such as cassava waste water [32], waste cooling oil [33], and soybean oil waste [34] have been used in the culture media for rhamnolipid production, which can reduce the cost of rhamnolipid production [32,35].

**Figure 1:**
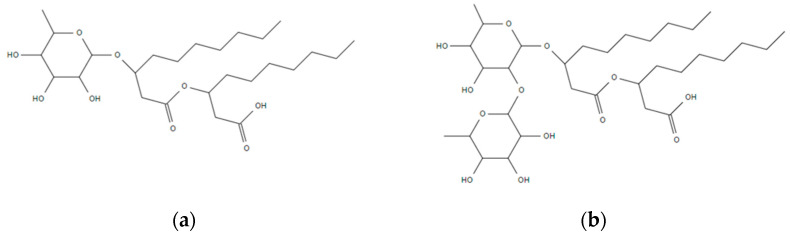
Chemical structures of (**a**) mono-rhamnolipid and (**b**) di-rhamnolipid.

Rhamnolipids consist of a mono- or di-rhamnose head group (i.e., mono-rhamnolipids or di-rhamnolipids, respectively), which is hydrophilic, and a hydrocarbon fatty acid chain as the hydrophobic tail [36,37]. The chemical structures of mono- and di- rhamnolipids [1] are shown in Figure 1. The rhamnolipids obtained from microorganisms are usually a mixture of rhamnolipid homologues. The chemical structure varies depending on the bacterial strain type, carbon source, and culture conditions [38,39]. As many as 28 different homologues of rhamnolipids have been reported, with the most common being Rha-C_10_-C_10_ and Rha-Rha-C_10_-C_10_ [40].

An improved understanding of the properties of biosurfactants under different conditions is essential for supporting their possible applications. One of the most important parameter in investigations concerning surfactants is the critical micellization concentration (CMC) [41], i.e., the concentration at which the surfactants start to form micelles in aqueous solution [36].

A compilation of rhamnolipid CMC data from articles published between 2000 and 2016 indicates the rhamnolipid CMC to be in the range 1.6–400 mg/L [42]. Since inconsistent terms were used to describe the purity of rhamnolipids in different publications, it is hard to make a reliable assessment of the purity based on these descriptions [42]. After summarizing data from 97 rhamnolipids of different purities and produced from different substrates, the authors concluded that the hydrophobicity of the carbon substrate that was used for the biosynthesis had the most significant influence on the CMC of the final rhamnolipid; the purity of the rhamnolipid also had a significant impact on CMC (generally the less pure the rhamnolipid, the higher the CMC). 

Another important property of surfactants is their adsorption behavior at solid–liquid interfaces. This plays a key role in controlling a series of interfacial processes for technological applications, for example, detergency, corrosion inhibition, dispersion of solids, mineral flotation, lubrication, and oil recovery [43]. Adsorption is also of widespread interest for potential applications in the fields of microelectronics, sensors, conductors, and thin insulators [43]. For some applications, such as enhanced oil recovery, adsorption of surfactants can be detrimental as it results in surfactant loss and reduced surfactant mobility; surfactant adsorption may even create new adsorption sites for hydrophobic compounds [44]. In other applications, strong adsorption can be desirable. 

Only a few types of biosurfactants have been studied in terms of their surfactant properties, and most publications focus on crude biosurfactants or mixtures of biosurfactant homologues (without reporting the composition). CMC values for biosurfactants have mainly been obtained from surface tension which, in the presence of impurities, is problematic [39]. There is a lack of attention on purified biosurfactants and physicochemical properties such as adsorption on solid surfaces.

We investigate here rhamnolipid micellization in aqueous solution and rhamnolipid adsorption on a model solid surface, with a focus on the role played by the difference between mono- and di-rhamnolipid chemical structures, rhamnolipid purity, and the salinity of the aqueous solution. The solution self-assembly and surface adsorption properties of rhamnolipids are compared with the corresponding properties of typical synthetic surfactants. An improved fundamental understanding of rhamnolipid interactions and association would be of great importance in designing more efficient, effective, and environment-friendly biosurfactant formulations for diverse applications.

## 2. Results and Discussion

### 2.1. Micellization in Aqueous Solution

**Figure 2:**
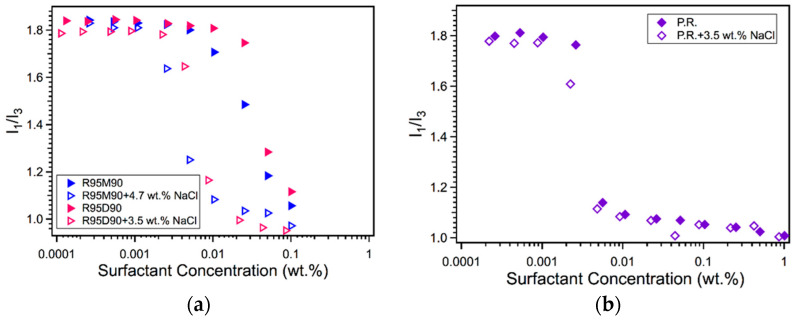
Pyrene fluorescence intensity I_1_/I_3_ ratios for (**a**) R95M90, R95D90, and (**b**) P.R. rhamnolipid in water and in aqueous NaCl solutions.

Plots of the pyrene fluorescence intensity I_1_/I_3_ ratio as a function of rhamnolipid aqueous solution concentration are shown in Figure 2 and the CMC values are summarized in Table 1. In addition to plain water, 3.5 wt.% (0.6 M) NaCl solution was utilized as a solvent in order to mimic seawater in connection to the potential application of biosurfactants in oil spill cleanup. 

Following the second method of CMC determination by pyrene fluorescence, the CMC values of R95M90 and R95D90 in water are 0.059 wt.% (1.17 mM) and 0.063 wt.% (0.97 mM), respectively. Due to the missing second rhamnosyl group, R95M90 is relatively less hydrophilic than R95D90 [45]. The difference between R95M90 and R95D90 CMCs is consistent with previous findings. For example, from surface tension measurements at 25 °C, Singh et al. [46] reported that purified R1 (purity not known) CMC is 150 mg/L (0.30 mM) and purified R2 (purity not known) CMC is 125 mg/L (0.19 mM). From surface tension measurements, Guo and Hu [47] also reported a higher CMC of R1 (0.11 mM, R1 purity ~80%) compared with the CMC value of R2 (0.07 mM, R2 purity ~75%). The CMC values of R95M90 and R95D90 obtained here are higher than those reported in the literature (1.6–400 mg/L, i.e., 1.6 × 10^−4^ wt.%–0.04 wt.%) [39,46,47,48]. The wide range of reported CMCs is probably due to the fact that the rhamnolipids mentioned in literature are of various purities, including cell-free culture broth, rhamnolipid mixtures with unknown compositions, etc. 

Comparing the purified rhamnolipids to crude P.R. rhamnolipid in terms of the CMC in water, the CMC of P.R., 0.006 wt.%, is around one order of magnitude lower than that of the purified R95. The lower CMC of P.R. rhamnolipid may be due to the different carbon source used [42] (the carbon substrate for P.R. is glucose, and for purified R95 is canola oil and/or vegetable oil substrate), or hydrophobic impurities present in the P.R. rhamnolipid. The crude P.R. rhamnolipids considered here were solvent extracted from the product of *Pseudomonas aeruginosa* cultured with glucose. The P.R. CMC is very close to the reported CMC 50 mg/L (~0.005 wt.%) of another crude rhamnolipid also produced from *Pseudomonas aeruginosa J4* with glucose as the carbon source and the solvent extracted [49]. 

The introduction of NaCl decreased the CMC of both R95M90 and R95D90 by ~80% (both reduced to 0.011 wt.%). Rhamnolipids are anionic surfactants due to the carboxylic groups, thus can be strongly affected by the solution pH and the presence of electrolytes [50,51]. According to the reported acid dissociation constant (pKa) values 5.5 [51] and 5.9 [52] for R1, and 5.6 [53] for R2, at a neutral pH, most of the rhamnolipid molecules are negatively charged, whereas, at acidic pH, most molecules are neutral [52]. The CMC of the negatively charged rhamnolipids was found to depend on the ionic strength, while the CMC of the protonated rhamnolipids did not [52]. Since it is reported that the natural pH of rhamnolipid solutions, including pure R1 (Rha-C_10_-C_10_), pure R2 (Rha-Rha-C_10_-C_10_), and their mixtures, is 6–6.8 [54], the rhamnolipid molecules in pure water or the aqueous NaCl solution in our study should all be negatively charged. Hence, the observed CMC reduction of rhamnolipids upon NaCl addition is attributed to diminishing repulsions between the rhamnolipid headgroups caused by the electrolyte. NaCl addition was also found to lower the CMCs of pure R1 (96%) and R2 (99%) (both from Jeneil Biosurfactant Co., Saukville, WI, USA) and to reduce the aggregate sizes [55].

NaCl addition also decreased the CMC of the crude P.R. rhamnolipid by about 20% (0.005 wt.%). Compared with the CMC changes of R95M90 and R95D90 (~80% reduction), the crude rhamnolipid exhibited a higher tolerance towards salinity, which may be due to the presence of impurities that affect CMC but are not affected by salt. 

As a comparison, the anionic surfactants sodium dodecyl sulfate (SDS) [56,57,58,59] and dioctyl sulfosuccinate sodium salt (Aerosol-OT, AOT) [58,60] were chosen as representative synthetic surfactants. Under the same conditions (in water, at 25 °C), the CMC values of SDS and AOT are 0.24 wt.% [61] and 0.12 wt.% [58], respectively. It is clear that rhamnolipids have a CMC in water at least 50% (60% if the molar concentration is used for comparison) lower than that of synthetic surfactants SDS or AOT. Salt addition reduced the CMC of SDS by 87% (in 1.6 wt.% NaCl solution) [57,62] and reduced the CMC of AOT by 92% (in 0.75 wt.% NaCl solution) [63], which is comparable to the CMC reduction (80%) of purified rhamnolipids. We note that the CMC values reported above were determined for simple systems, e.g., surfactant in plain water, or surfactant in aqueous salt solution. In practical applications where other ingredients are present, the onset of micelle formation may be affected by the system.

**Figure 3:**
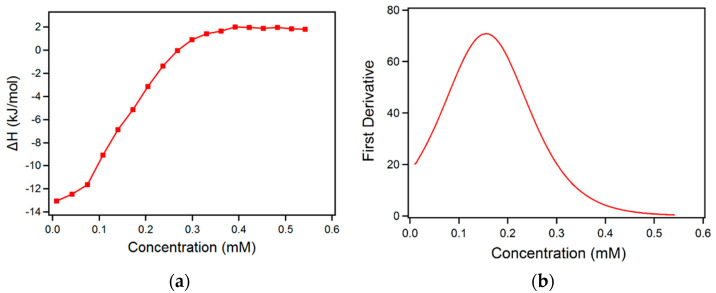
(**a**) ΔH of injection per mole of injected R95D90 in aqueous solution plotted as a function of the R95D90 concentration in the calorimeter cell; (**b**) first derivative of the ΔH vs. surfactant concentration.

In order to shed further light on the micellization of the rhamnolipids in plain water, micellar R95D90 aqueous solution was successively titrated into water with ITC. ITC is a very useful technique to characterize a demicellization process from a thermodynamic point of view [64], and can also be used to determine CMC. The CMC can be determined from the first derivative of the heat versus concentration plot. As the CMC is reached, the observed enthalpy change decreases gradually and, for the last several titrations, the micelles stop dissociating into unimers, and only dilution of the micelles takes place. During the dilution process of the concentrated surfactant micellar solution, the heat released or consumed in each dilution step was recorded by ITC. The enthalpy difference between the end and the start of the micelle dissociation is the enthalpy of micellization $\Delta H_{mic}\;$ [64]. To calculate $\Delta H_{mic},$ linear fits were obtained for the first and for the last several points in Figure 3a. The points at which the fits diverged from the enthalpy curve were chosen as the start/end point of the micelle dissociation [64]. Figure 3b shows the first derivative of this titration curve. The CMC (maximum of the first derivative) is located at a concentration of 0.156 mM (0.010 wt.%). This CMC value is in the range of the reported CMC, but lower than the value from our fluorescence data. Since the affinity of pyrene to micelles depends strongly on the type of surfactant [65], during the fluorescence measurement, pyrene might not be adequately solubilized into micelles due to the strong polarity of rhamnolipid headgroups with relatively short hydrophobic tails, resulting in a higher CMC value from fluorescence data. The CMC of R95D90 determined with ITC (0.010 wt.%) is close to a reported CMC of R2, 0.007 wt.% (0.110 mM), at 25 °C determined by ITC [66], but the solvent is different (150 mM NaCl, 5 mM HEPES, pH 7.4).

The tendency of rhamnolipids to associate in aqueous solution can be rationalized by the thermodynamic parameters of the micellization process. The Gibbs free energy of micellization for ionic surfactants can be obtained from [59,67]

(1) $$\Delta G_{\mathrm{mic}}=(2\;-\;\alpha )\;\mathrm{RT}\;\ln\left(\mathrm{CMC}\right)$$

where *α = m/n* is the degree of micelle ionization (with *n* being the micelle aggregation number and *m* the number of counterions per micelle dissociated from the micelle). In Equation (1), the CMC is expressed in mole fraction units. With both $\Delta H_{mic}$ and $\Delta G_{mic}\;$ known (in our case at T = 25 °C), the entropy of micellization $\Delta S_{mic}$ can be calculated from the Gibbs Equation:

(2) $$\Delta G_{\mathrm{mic}}=\Delta H_{\mathrm{mic}}\;-\;T\Delta S_{\mathrm{mic}}$$

The degree of micelle ionization, *α*, for the rhamnolipids is not known, but is expected to be lower than that of SDS or AOT. The rhamnolipids are anionic surfactants due to the presence of the carboxylate groups in their structure; however, at the neutral pH of the solutions studied here, the degree of H^+^ dissociation from the micelle is estimated to be low. The degree of micelle ionization, *α*, for SDS and AOT (both having Na^+^ counterions) at their CMC, at 25 °C, obtained by a variety of methods is ~0.23 [57,59] and ~0.33 [58,60], respectively. Considering a R95D90 rhamnolipid degree of micelle ionization between 0.10–0.25, based on Equation (1), the Δ*G_mic_* of R95D90 micellization in water can have a lower value of −60.2 kJ/mol (for *α* = 0.10) and an upper value of −55.4 kJ/mol (for *α* = 0.25), with the negative value consistent with spontaneous micelle formation when the concentration reaches the CMC. Both values are considerably lower than the free energy of micellization −38.8 kJ/mol for SDS [57] and −32.8 kJ/mol for AOT [58], confirming the higher tendency of rhamnolipids to form micelles compared to the synthetic surfactants. The Δ*H_mic_* of R95D90 determined by our ITC measurements is 12.6 kJ/mol. This positive enthalpy value means that the transfer of a R95D90 molecule from the aqueous solution to a micelle is an endothermic and enthalpically unfavorable process [66]. The entropy term *T*Δ*S_mic_* of R95D90 is calculated to be 68 kJ/mol–72.8 kJ/mol (based on the above values of Δ*G_mic_*). This positive entropy of micellization is commonly explained by the hydrophobic effect, primarily arising from the strong attractive forces between the water molecules, which have to be disturbed when the amphiphile is dissolved.

### 2.2. Adsorption on Solid Surfaces

Hydrophilic surfaces are highly desirable in many applications, e.g., anti-fogging, anti-fouling, detergent-free cleaning, oil and water separation [68], water treatment, and biomedical applications [69], etc. Hydrophilic solid materials include quartz [70], kaolin [71], diatomite [71], fly ash [71], calcite, fluorite, apatite, magnetite, highly oxidized coal, etc. [72] The knowledge of surfactant adsorption on hydrophilic surfaces is relevant to applications involving these minerals. The reported contact angle of the QCM-D gold sensors is only 11° [73], thus we selected the gold surface as a model hydrophilic surface to study rhamnolipid adsorption. The concentration of each pure rhamnolipid sample (0.15 wt.%) or P.R. rhamnolipid sample (0.10 wt.%) used in the adsorption experiments is much higher than its CMC value in water. 

**Figure 4:**
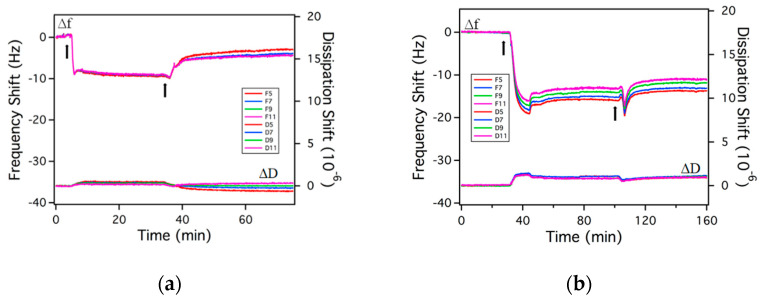
Δ*f* and Δ*D* over time of a gold sensor surface responding to exposure to (**a**) 0.15 wt.% R95M90 aqueous solution and (**b**) 0.15 wt.% R95M90 in 3.5 wt.% NaCl aqueous solution. The first arrow indicates the time when the rhamnolipid solution was injected and the second arrow indicates the time when solvent rinsing was initiated. *Fi* and *Di* shown in the insert are the frequency and dissipation at *i^th^* overtone, respectively.

**Figure 5:**
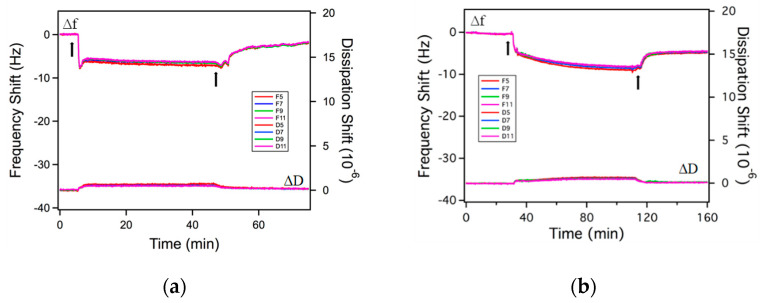
Δ*f* and Δ*D* over time of a gold sensor surface responding to exposure to (**a**) 0.15 wt.% R95D90 aqueous solution and (**b**) 0.15 wt.% R95D90 in 3.5 wt.% NaCl aqueous solution. The first arrow indicates the time when the rhamnolipid solution was injected and the second arrow indicates the time when solvent rinsing was initiated. *Fi* and *Di* shown in the insert are the frequency and dissipation at *i*th overtone, respectively.

**Figure 6:**
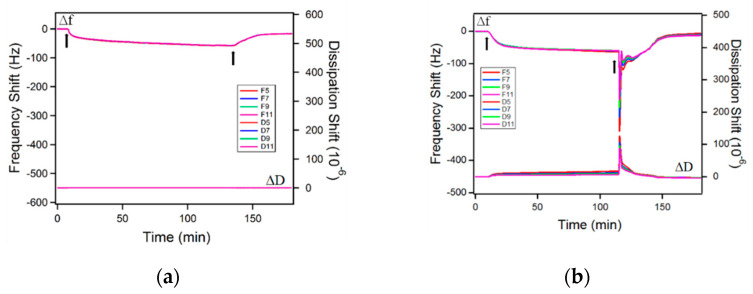
Δ*f* and Δ*D* over time of a gold sensor surface responding to exposure to (**a**) 0.1 wt.% P.R. rhamnolipid aqueous solution and (**b**) 0.1 wt.% P.R. rhamnolipid in 3.5 wt.% NaCl aqueous solution. The first arrow indicates the time when the rhamnolipid solution was injected and the second arrow indicates the time when solvent rinsing was initiated. *Fi* and *Di* shown in the insert are the frequency and dissipation at *i*th overtone, respectively.

The adsorption of rhamnolipids on gold surfaces is shown in Figure 4, Figure 5 and Figure 6. During the measurement, both the frequency and dissipation shifts are monitored and recorded in real time. All adsorption experiments involved three steps: (i) Rinsing of the gold surface with the solvent to set the baseline for the measurement; the frequency and dissipation shifts are zero in this process; (ii) adsorption of the rhamnolipids from solution on the gold surface, causing a negative frequency shift and a positive dissipation shift; (iii) after the frequency and dissipation shifts reach a plateau, rinsing of the adsorbed layer with the solvent, during which surfactant desorbs from the gold surface, thus the frequency shift increases, and the dissipation shift decreases. In the QCM figures presented next, the first arrow indicates the time when the rhamnolipid micellar solution was injected, while the second arrow denotes the time when rinsing with the solvent was initiated.

The model to calculate the adsorbed surfactant amount is selected based on the adsorbed layer softness. The equilibrium Δ*D*/Δ*f* ratio (Δ*D*/Δ*f* ratio at equilibrium adsorption) is often used as an indication of the layer softness [74]. In plain water as a solvent, the Δ*D*/Δ*f* ratios of both R95M90 and R95D90 are very small, indicating that the adsorbed layers are rigid. The Sauerbrey relation (Equation (4), Section 3) is applicable in these cases to calculate the areal mass of adsorbed molecules [75]. After rinsing with solvent (plain water), Δ*D* and Δ*f* did not return to zero, meaning that there was still rhamnolipid remaining on the surface, thus the adsorption of both surfactants is partially irreversible. The adsorbed amounts before and after solvent rinsing are summarized in Table 2. R95D90 adsorbed less than R95M90 before and after rinsing, possibly due to steric or packing constraints of the larger R95D90 di-rhamnose headgroups. Very little information is available in the literature on the morphology of rhamnolipid layers adsorbed on a solid surface, so the structure of rhamnolipids at the aqueous solution–air interface is used here to infer the structure of the adsorbed layer on a solid surface. The area that each surfactant molecule occupied on the gold surface was calculated from Equation (9) (Section 3). In plain water, the area per adsorbed R95M90 molecule before rinsing was 0.47 nm^2^, almost half of the area occupied by R1 in the saturated monolayer at the water–air interface (0.826 nm^2^ [39], calculated with the maximal Gibbs surface excess concentration obtained from surface tension data), suggesting a possible bilayer of R95M90 formed on the gold surface. At 0.85 nm^2^/molecule, R95D90 surfactant molecules take up about double the space as the R95M90 molecules do in plain water (0.47 nm^2^).

To elucidate the structure of the adsorbed layer, we invoke the critical packing parameter (CPP) value as a comparison to the structure of the aggregates in solution. CPP is defined as [76]:

(3) $$\mathrm{CPP}=\frac{v_{tail}}{l_{tail\;}a_{0}}$$

where *υ_tail_* is the molecular volume (nm^3^) of the hydrophobic portion of the surfactant, *l_tail_* is the maximum length (nm) of a fully extended hydrocarbon chain with $l_{tail}=0.15+0.127n_{c}$, and $v_{tail}=0.027\left(n_{c}+n_{Me}\right)$ ($n_{c}\;$ is the total number of carbon atoms per chain, and $n_{Me}$ is the number of methyl groups). The hydrophobic tail properties of RLs are calculated as *l_tail_* = 0.912 nm, *υ_tail_* = 0.432 nm^3^. In plain water, the CPP value for R95M90 is 1.0, suggesting that R95M90 favors a planar bilayer. With the density of the adsorbed layer of rhamnolipids equal to 1.06 g/cm^3^, the R95M90 layer thickness in water is calculated to be 1.7 nm (based on Equation (5), Section 3), almost twice the surfactant tail extended length, 0.912 nm, further supporting the assessment that R95M90 adsorbed as a bilayer on the gold surface from water. The CPP values for R95D90 in plain water and in 3.5 wt.% NaCl aqueous solution are both close to 0.6, which is in the range 1/2 < CPP < 1. The R95D90 layer thickness in both cases (1.2 nm in plain water and 1.4 nm with salt) is larger than the tail extended length, but less than twice the extended chain length, which indicates thicknesses between those of a monolayer and a bilayer, with the most possible scenario being that of R95D90 micelles adsorbed on the gold surface.

The influence of rhamnolipid purity on the adsorption behavior was also investigated. The adsorbed layer of P.R. rhamnolipid is also rigid (equilibrium Δ*D*/Δ*f* = 0.01) and the Sauerbrey Equation is applicable. The calculated area per molecule occupied on the gold surface is as small as 0.1 nm^2^/molecule based on the large adsorbed amounts, suggesting the formation of a rather thick layer on the gold surface, which might be attributed to hydrophobic impurities present in the P.R. rhamnolipid sample.

**Figure 7:**
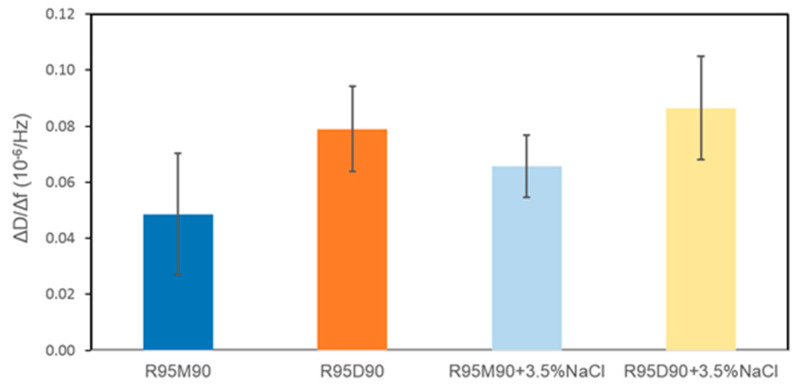
Equilibrium Δ*D*/Δ*f* values of 0.15 wt.% R95M90 and R95D90 adsorbed layers on gold surfaces from water and 3.5 wt.% NaCl aqueous solution.

Similar to the case of plain water, the adsorption of purified rhamnolipids from 3.5% NaCl aqueous solutions is partially irreversible. Although Δ*D*/Δ*f* ratios for both R95M90 and R95D90 increased slightly, they are still in the 0.04–0.10 range (see Figure 7), indicating that the adsorbed layers are rigid. The adsorbed amount of both surfactants increased since the repulsion between the rhamnolipid headgroups was reduced by salt. R95D90 still adsorbed less than R95M90 due to larger R95D90 di-rhamnose headgroups. The adsorbed amount of R95M90 increased more (48%) upon electrolyte addition compared to R95D90 (17%), which is in accordance with the report by Helvaci et al. [55] that states that the effect of the reduction in the repulsive interactions is stronger in the case of the more hydrophobic R1 molecules. In the presence of NaCl, the irreversible adsorbed amounts for R95M90 and R95D90 also increased, indicating a stronger binding to the gold surface due to the screening of electrostatic repulsion, consistent with the effects of salt on ionic surfactant binding to surfaces [77,78]. Helvaci et al. [55] also calculated the coefficient of elasticity for R1 and R2 at the CMC, and concluded that R1 formed a more rigid layer at the air–solution interface than R2 with increasing NaCl concentration, which is in agreement with our results that show that the equilibrium Δ*D*/Δ*f* (softness) of R95M90 is slightly lower than that of R95D90.

**Figure 8:**
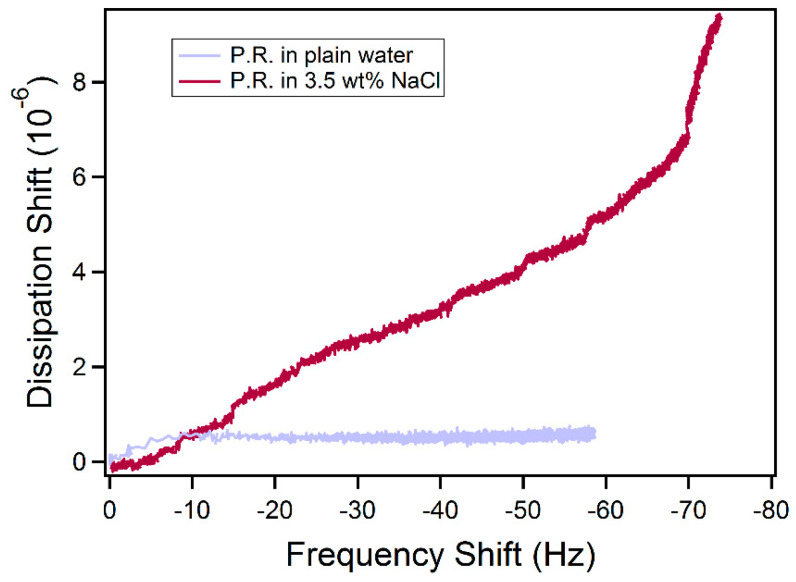
Variation of dissipation shift Δ*D* vs. frequency shift Δ*f* (5th overtone) during the adsorption of P.R. rhamnolipid on a gold sensor surface from aqueous solution and from 3.5 wt.% NaCl aqueous solution.

Salt addition promoted the adsorption of the P.R. rhamnolipid (the amount of P.R. adsorbed from brine is twice as much as that from plain water) and adsorption was still partially reversible. However, after adding salt, the viscoelasticity of the adsorbed film changed according to the large equilibrium Δ*D*/Δ*f* ratio (0.13) observed for P.R, and we applied the viscoelastic Voigt model here for the adsorbed mass calculation (considering the density and viscosity of salt water at 25 °C [79,80]). The Δ*D* vs. Δ*f* plot reveals the conformation of the adsorbed layers: a straight line suggests the buildup of a homogeneous layer, while a curved profile may indicate variations in the conformation with the degree of coverage [81]. In plain water, P.R. rhamnolipid adsorbed as rigid layer during the whole adsorption process according to the low Δ*D* value; however, in 3.5 wt.% NaCl aqueous solution, the adsorbed layer became more viscoelastic. In the presence of NaCl, two different slopes of Δ*D* vs. Δ*f* plot are observed (Figure 8). The slope between −70 Hz and −74 Hz is higher than that corresponding to the 0 to −70 Hz regime, showing that the film became softer as more P.R. rhamnolipids accumulated on the surface after Δ*f* reached −70 Hz. The percentage of irreversible adsorbed P.R. rhamnolipids on the gold surface did not increase with NaCl addition, indicating that NaCl has less influence on P.R. rhamnolipid adsorption compared to salt effects on R95M90 and R95D90 adsorption, most probably owing to the P.R. impurities, which are not affected by salt.

Very few studies of rhamnolipid adsorption on solid surfaces have been published. We are aware of only one article on rhamnolipid adsorption probed by QCM-D. The rhamnolipids used were a mixture of R1 and R2 in the ~1:5 ratio [74]; this composition (~83% R2) is close to the R95D90 sample considered here. The adsorption behavior of rhamnolipids was investigated on cranberry proanthocyanidin (CPAC)-coated TiO_2_ surfaces, and was found within the Sauerbrey regime, meaning that the film is rigid [74], and the adsorbed mass can be calculated directly from Δ*f* (Table 3). Similar to our study, irreversible adsorption occurred on the CPAC surface. Compared with R95D90 on gold, more rhamnolipid molecules remained on CPAC after rinsing; the possible reason being a higher affinity between the rhamnolipid molecules and the CPAC functional groups (information on the characterization was not reported in [73]). The ratio of Δ*D*/Δ*f* at equilibrium adsorption on CPAC was also calculated as an indication of the viscoelasticity of the equilibrium layer [74]. At low rhamnolipid concentrations (below 40 μM), the Δ*D*/Δ*f* ratio is quite low, whereas at high concentrations (higher than 40 μM), this ratio exhibited a marked increase, suggesting a change in adsorbed form from rhamnolipid molecules to viscoelastic rhamnolipid micelles [74]. A few publications have considered rhamnolipid Adsorption on soil in batch adsorption experiments. Noordman et al. [44] investigated the adsorption of R1 and R2 (six congeners) mixtures on two sandy soils; the adsorbed amount was determined from the difference in the rhamnolipid concentration in the aqueous phase before and after adsorption. They concluded that more hydrophobic rhamnolipid components are preferentially adsorbed. For another rhamnolipid mixture (eight congeners), the more hydrophobic congeners also showed preferential adsorption to soil (76% sand, 21% silt, and 3% clay) [82]. Renfro et al. [83] reported that the formation of rhamnolipid (composition not mentioned) micelles prevented rhamnolipid adsorption to soil (40% clay, 30% sandy clay, and 30% highly clayey sand) above the CMC.

## 3. Materials and Methods

### 3.1. Rhamnolipids

In summary, 95% pure rhamnolipid containing 90% mono-rhamnolipid (R95M90) (Sigma, St. Louis, MO, USA, AGEA Technologies), 95% pure rhamnolipid containing 90% di-rhamnolipid (R95D90) (Sigma, St. Louis, MO, USA, AGEA Technologies), and *Pseudomonas aeruginosa* rhamnolipid (P.R.) crude extract (University of Ulster) were used as received. In what follows, “R95M90” and “R95D90” refer to the mono-RL and di-RL samples from Sigma used in our study, while “R1” and “R2” refer to mono-RL and di-RL, respectively, as reported in the literature. In converting from mass to molar concentrations, we used 504.7 g/mol and 650.8 g/mol as the M.W.s for R95M90 and R95D90 samples, respectively.

### 3.2. Other Chemicals

Milli-Q grade (18 MΩ·cm) water was used as the solvent. Sodium chloride (NaCl) (>99%, EMD Millipore Sigma, Burlington, MA, USA) aqueous solution was prepared with Milli-Q water. Pyrene (>99%, Sigma, St. Louis, MO, USA) and ethanol (200 proof, Decon labs, Inc., King of Prussia, PA, USA) were used in the fluorescence spectroscopy measurements. Ammonium hydroxide (25% in water, Sigma Aldrich, St. Louis, MO, USA) and hydrogen peroxide (30% in water, Fisher Scientific, Hampton, NH, USA) were used for cleaning the gold sensors prior to each QCM-D measurement.

### 3.3. Sample Preparation

For the fluorescence spectroscopy measurements, 0.1 wt.% R95M90 or R95D90 aqueous stock solution, and 1.0 wt.% crude P.R. rhamnolipid aqueous stock solution were first prepared, and then mixed on a rotator for at least 24 h. Rhamnolipid aqueous solution samples of various concentrations in the range 0.0001–1.0 wt.% were prepared by dilutions from the stock solutions. Pyrene solution was prepared by dissolving 0.01 g pyrene in 50 mL ethanol and mixed for at least 24 h [84,85]. A total of 2 µL of the as-prepared pyrene solution was added to each aqueous solution sample and then mixed at room temperature. The fluorescence experiments were carried out within two days of pyrene addition to the rhamnolipid aqueous solutions. To prepare surfactant in NaCl aqueous solutions, a small mass of concentrated NaCl aqueous solution was added to each surfactant solution sample in order to achieve a salt content of 3.5 wt.% (simulating sea water salinity) without significantly altering the original surfactant concentration. Moreover, 4.7 wt.% NaCl was used in certain samples.

For samples used in ITC or QCM-D (refer to the Methods Section), a specific mass of surfactant was mixed with the aqueous solvent to obtain a specific total mass of sample, followed by at least 10 h of rotation at room temperature to allow for equilibration prior to the measurement. 

### 3.4. Fluorescence Spectroscopy

The fluorescence data for the determination of CMC and micropolarity were obtained with a Hitachi F-2500 Fluorescence Spectrophotometer (Tokyo, Japan). An excitation wavelength of 335 nm was used, and data were recorded between 340 and 460 nm at 25 °C. The determination of CMC by pyrene was based on the solvent dependence of the vibrational band intensities in the pyrene monomer fluorescence [64]. Pyrene is a hydrophobic molecule exhibiting five characteristic vibronic bands in the fluorescence spectrum. The bands vary in intensity as pyrene interacts with solvents of different polarities to produce different intensity profiles when excited from the ground state. The five peaks are referred to as I_1_-I_5_, from shorter to longer wavelengths. The intensity ratio I_1_/I_3_ increases with increasing polarity of the environment where pyrene is located [65,84,86]. Below CMC, pyrene senses the polar environment of the aqueous solvent, and the I_1_/I_3_ ratio is relatively high. Above CMC, the hydrophobic pyrene molecules are solubilized in the interior of micelles; hence, the pyrene senses a less polar environment and the I_1_/I_3_ ratio decreases. This results in a sigmoidal decrease in 5the I_1_/I_3_ ratio around the CMC in an I_1_/I_3_ versus log (surfactant concentration) plot [65].

Two methods can be used for CMC determination from pyrene I_1_/I_3_ data [41,87]: (i) Calculate the first derivative of the I_1_/I_3_ ratio versus concentration, and the concentration at which the derivative is minimum is the CMC value (CMC_1_); this is typical of nonionic surfactants; (ii) the concentration where the line fitting the decreasing portion of the plot intersects with the lower limit plateau line is identified as CMC (CMC_2_); this method is typically applicable in ionic surfactants [41].

### 3.5. Isothermal Titration Calorimetry

Isothermal Titration Calorimetry (ITC) (iTC200 MicroCal, Malvern, Westborough, MA, USA) was used to determine the thermodynamic parameters of the micellization [66] and the CMC. R95D90 aqueous solution at a much higher concentration than the CMC was successively injected with 1.5 μL per injection to the Milli-Q water in the ITC cell, and the heat release or uptake during each injection was recorded. The initial injections of the concentrated surfactant solution to the ITC cell resulted in a solution with a concentration below CMC, and micelles were disassembled into individual surfactant molecules (unimers) [88]. As more concentrated surfactant solution was added, the demicellization heat decreased. The CMC can be determined from the first derivative of the heat versus concentration plot. 

### 3.6. Quartz Crystal Microbalance with Dissipation (QCM-D)

The adsorption experiments were carried out with a model E4 QCM-D instrument and QSX301 gold sensors (Q-Sense, Biolin Scientific, Gothenburg, Sweden). QCM-D is a very useful and sensitive tool to measure mass adsorption/desorption and viscoelastic behavior of the adsorbed film [89]. The adsorption isotherms, film thickness, and adsorption/desorption kinetics can be obtained by QCM-D via analyzing the changes of mass and viscoelastic characteristics of the film [90,91].

The sensor is a thin plate of a piezoelectric quartz crystal, sandwiched between a pair of gold electrodes. The quartz crystal can oscillate at a specific frequency when a voltage is applied. For rigid adsorbed layers, the frequency (*f*) will decrease as mass is adsorbed onto the surfaces of the crystal. The mass change can be calculated from the frequency change (Δ*f*) using the Sauerbrey Equation [92]:

(4) $$\Delta m=-C\frac{1}{n}\Delta f$$

where *C* is the sensitivity constant, which is characteristic of the specific quartz crystal; *n* is the overtone number (*n* = 1, 3, 5,…); and Δ*m* is the mass adsorbed on the crystal surface [93]. It should be noted that the Sauerbrey relation is only valid when the following conditions are fulfilled [93,94]: (i) The mass adsorbed is evenly distributed over the crystal; (ii) Δ*m* is much smaller than the mass of the crystal itself; and (iii) the mass adsorbed is rigidly attached to the surface. 

Based on these assumptions, it is also possible to estimate the adsorbed layer thickness (*d_eff_*):

(5) $$d_{\mathrm{eff}\;}=\frac{\Delta m}{{A\;\rho }_{\mathrm{eff}}}$$

where *ρ_eff_* is the effective density of the adsorbed layer and *A* is the active area of the crystal (in our case, 0.785 cm^2^) [95].

If the adsorbed layer on the quartz crystal is not rigid, it will not fully couple to the oscillation of the crystal, and the mass on the surface would be underestimated by the Sauerbrey Equation, thus viscoelasticity must be taken into consideration. The simultaneous measurements of changes in both resonance frequency and energy dissipation of QCM-D enables its application for studying soft films. The dissipation factor *D* is proportional to the power dissipation in the oscillatory system and can provide valuable information about the film rigidity [90]:

(6) $$D=\frac{E_{\mathrm{dissipated}}}{{2\pi E}_{\mathrm{stored}}}$$

(7) $$\Delta D=\frac{1}{\rho_{q}t_{q}}\sqrt{\frac{\rho_{l}\eta_{l}}{2\pi f}}$$

where *E_dissipated_* is the energy dissipated during one oscillation and *E_stored_* is the energy stored in the oscillating system. Equation (7) describes the dissipation shift (Δ*D*), *ρ_q_* and *t_q_* are the density and thickness of the crystal quartz sensor, respectively, and *ρ_l_* and *η_l_* represent the density and viscosity of the fluid, respectively [75,93]. Therefore, any coupling between the bulk medium and crystal will affect the dissipation. This coupling, in turn, will affect the viscoelastic properties of the layer adsorbed to the surface. The response of a freely oscillating sensor, which is vibrated at its resonance frequency, can be recorded to measure the dissipated energy. Voigt modeling (Equation (8)) is the most common viscoelastic model used for estimating structural properties of adsorbed soft film in QCM-D measurements [96]. The Voigt model represents a solid undergoing reversible viscoelastic strain:

(8) $$G^{*}{=G}^{\prime }{+iG}^{\prime \prime }{=\mu }_{1}{+i2\pi f\eta }_{1}$$

where $G^{*}$, $G^{\prime }$, and $G^{\prime \prime }$ are the complex shear modulus, storage modulus, and loss modulus, respectively; *µ, f,* and, *η* are the elasticity, frequency, and shear viscosity coefficient, respectively [96,97]. The incorporation of the viscoelastic model into the Q-Sense software QTools following the measurement at multiple overtones can help characterize the adsorbed film in detail. The layer viscosity, shear modulus, and adsorbed mass may be extracted for soft adsorbed layers with certain assumptions on the layer density and fluid properties. 

To be specific, if Δ*D* is less than 2 × 10^−6^ or if Δ*D*/Δ*f* is very small, the Sauerbrey relation can be used to obtain the adsorbed mass [75]. For a large Δ*D*, the film is not rigid and the viscoelastic model can be utilized for data analysis. During the adsorption process, *f* and *D* shifts at various overtones (5th, 7th, 9th, and 11th) are collected in real time. For a rigid film, mass change is only related to the Δ*f* value (Sauerbrey Equation), and Δ*m* is the average of the mass changes at different overtones of the sensor. However, in a viscoelastic system, each harmonic is unique; thus, *f* and *D* shifts at different overtones of one sensor are included in the modeling to determine Δ*m*.

Assuming the soft film is homogeneous, the film thickness can also be calculated via Equation (5). Furthermore, the area per molecule of adsorbed surfactant, *a_o_* (nm^2^/molecule,) that occupies the sensor surface can be obtained from Equation (9) using the mass adsorbed, surfactant molecular weight (M.W.), and Avogadro’s number (*N_Avogadro_*):

(9) $$a_{o}=\frac{1}{\left(\Delta m\frac{\mathrm{ng}}{{\mathrm{cm}}^{2}}\right)\left(10^{-9}\frac{g}{\mathrm{ng}}\right)\left(\frac{1}{M.W.}\times \frac{\mathrm{mol}}{g}\right)\left(N_{\mathrm{Avogadro}}\frac{\mathrm{molecule}}{\mathrm{mol}}\right)}\left(\frac{10^{14}{\mathrm{nm}}^{2}}{{\mathrm{cm}}^{2}}\right)$$

All QCM-D measurements were carried out at 25 ± 0.05 °C. The flow rate was 0.1 mL/min for all the liquids. Initially the QCM-D modules were flushed with the solvent to obtain a stable baseline. Then, the surfactant solution flowed through the modules. When the frequency reached a plateau, solvent was flushed over the sensors. The data from overtone frequencies 5, 7, 9, and 11 were analyzed for each sensor. Experiments were performed on three sensors for each adsorption measurement, and the data are reported with a standard error. Prior to use, each gold sensor was cleaned following the Q-sense Cleaning and Immobilization Protocols to remove possible organic material and dust contaminants, and to obtain reproducible results. The gold sensors were first placed in an UV/ozone chamber for a 10-min treatment. They were then submerged in a 5:1:1 mixture of Milli-Q water, ammonium hydroxide (25% in water,) and hydrogen peroxide (30% in water) at 75 °C for 5 min. Subsequently, they were rinsed with Milli-Q water, dried with nitrogen gas, and again placed in the UV/ozone chamber for a 10 min treatment. 

## 4. Conclusions

Rhamnolipids can be promising substitutes for the synthetic surfactants that are currently utilized in many applications, including bioremediation and oil spill cleanup. However, there is a lack of information on rhamnolipid micellization in aqueous media and adsorption on solid surfaces, which hinders their efficient utilization. This work discusses the effects of chemical structure (mono- and di-rhamnolipids), rhamnolipid purity, and salinity on the rhamnolipid micellization and adsorption properties.

The difference between the chemical structures of mono-rhamnolipid R95M90 and di-rhamnolipid R95D90 (which has a second rhamnosyl group) renders R95M90 more hydrophobic. R95M90 requires more molecules to shield the lipophilic chain from aqueous solution since it has a smaller hydrophilic headgroup than R95D90, resulting in a slightly higher CMC of R95M90 in plain water. The larger R95D90 di-rhamnose headgroups also lead to a lower adsorbed mass than that of R95M90 on a model hydrophilic (gold) surface due to packing constraints. Both R95M90 and R95D90 adsorbed as rigid films and their adsorption was found to be irreversible to some extent. 

The presence of NaCl in water reduced the repulsion between headgroups of the negatively charged purified rhamnolipids; hence, the CMC values decreased and the amount of both surfactants adsorbed on the gold surface increased. The adsorbed amount of R95M90 increased more compared with R95D90, indicating that the effect of the reduction in the repulsive interactions are stronger for the more hydrophobic R95M90 molecules. The softness of the adsorbed R95M90 film is slightly lower than that of the R95D90 film.

The purity of rhamnnolipids has an influence on the micellization in aqueous solutions and adsorption properties on a solid surface. The crude P.R. rhamnolipid has a one order of magnitude lower CMC in plain water in comparison with the purified rhamnolipids. The crude rhamnolipid showed a higher tolerance towards salinity compared with the CMC change of R95M90 and R95D90; these observations are consistent with the presence of hydrophobic impurities in P.R. rhamnolipid. In plain water, similar to the purified rhamnolipids, P.R. formed a rigid film on the gold surface. However, in NaCl aqueous solution, the adsorbed layer of R95M90 or R95D90 was still rigid, whereas the P.R. layer was much softer, and the increased amount of P.R. rhamnolipid adsorbed on the surface was much higher. The rhamnolipid biosurfactants have a lower CMC than typical synthetic ionic surfactants in both plain water and aqueous NaCl solutions. 

For the first time, this work presents the effects of the chemical structure, purity, and salinity on rhamnolipid adsorption on a solid surface. There is only one report available on rhamnolipid adsorption on a CPAC-modified surface and a few on rhamnolipid adsorption in soil. A fundamental understanding of rhamnolipid solution self-assembly and surface adsorption properties is of great importance for the design of more effective and environmentally sustainable products for future applications. 

## Figures and Tables

**Table 1:** CMC values of R95M90, R95D90, and P.R. rhamnolipids in water and in NaCl aqueous solutions, determined from pyrene fluorescence by the linear intercept of the pyrene I_1_/I_3_ vs. log (surfactant concentration) plot.

Surfactant	NaCl (wt.%)	CMC (wt.%)	CMC (mM)
R95M90	0	0.059	1.17
	4.7	0.011	0.22
R95D90	0	0.063	0.97
	3.5	0.011	0.17
P.R.	0	0.006	0.10
	3.5	0.005	0.09

**Table 2:** Adsorption properties of rhamnolipids adsorbed from aqueous solutions on gold surfaces during QCM-D experiments.

Surfactant	NaCl (wt.%)	Model	Areal Mass Adsorption (ng/cm^2^)	Area Per Molecule before Rinsing (nm^2^/molecule)	Average Area per Molecule after Rinsing ^a^ (nm^2^/molecule)	Irreversibly Adsorbed Mass ^b^ (ng/cm^2^)
0.15 wt.% R95M90	0	Sauerbrey	178 ± 24	0.47	1.07	79 ± 8 [44%]
	3.5	Sauerbrey	263 ± 28	0.32	0.45	185 ± 54 [70%]
0.15 wt.% R95D90	0	Sauerbrey	127 ± 18	0.85	2.99	36 ± 16 [28%]
	3.5	Sauerbrey	148 ± 14	0.73	1.34	81 ± 15 [55%]
0.1 wt.% P.R.	0	Sauerbrey	1002 ± 49	0.10	0.32	297 ± 40 [30%]
	3.5	Viscoelastic	1915 ± 398	0.05	0.27	352 ± 19 [18%]

^a^ area values were calculated by assuming a full coverage on the surface; ^b^ the data in brackets are the % difference between the initial adsorbed mass and the adsorbed mass after rinsing.

**Table 3:** Frequency shift data and mass data of R95D90 or P.R. rhamnolipid adsorption on gold surface and of rhamnolipid (R1:R2 = 1:5) adsorption on CPAC-coated surface.

	0.15 wt.% R95D90 in Water on Gold (Our Data)	0.10 wt.% P.R. in Water on Gold (Our Data)	Rhamnolipid in PBS on CPAC-Coated Surface [74]
Δ*f* after adsorption	−7 Hz (126.8 ng/cm^2^)	−58 Hz (1018 ng/cm^2^)	~−60 Hz (~1062 ng/cm^2^)
Δ*f* after desorption	−2 Hz (36.1 ng/cm^2^) [28%]	−17 Hz (303 ng/cm^2^) [30%]	~−30 Hz (~531 ng/cm^2^) [50%]

Note: The data in brackets are the % difference between the initial adsorbed mass and the adsorbed mass after rinsing.

## Data Availability

Data supporting reported results can be obtained by contacting Prof. Marina Tsianou.
